# Convulsions in Children Treated with Large Doses of Sulphate of Quinine

**Published:** 1859-02

**Authors:** E. H. W. Hunter

**Affiliations:** Louisville, Ga.


*Convulsions in Children treated with large doses of Sulphate of Quinine.* By E. H. W. HUNTER, M. D., Louisville, Ga.

Prof. J. HARRIS—*Dear sir:* In compliance with your request, I proceed to write a short account of my experience in the treatment of

Convulsions in Children with Sulphate Quinine in *large doses.* It will be imperfect and lacking in that minuteness of detail which should characterize the report of all cases of disease; this defect is unavoidable from the fact that I write from memory, except in the the particulars of age and dates. The effects of the remedy were so happy and prompt in the first cases in which I used it, that an impression was made on my mind at the time, which has enabled me to re_ member the most important particulars in each case. The circumstances which first induced me to adopt the treatment, will be given in the following case, which though not one of convulsions, may, I think, without any impropriety, be reported in an article on the subject.

Case 1. Dentition, I was requested, in the absence of the family physician, on the morning of the 8th Sept., 1856, to visit the house of Mr. K., living two miles in the country, and lance the gums of his daughter Julia, aged 6 months and 19 days, light hair, blue eyes and fair complexion. Found her with some fever and the gums over the upper incisor teeth very much swollen. Learned she had had fever since the day before. After scarifying her gums, advised a dose of castor oil, as her bowels were constipated. Feeling very confident she would have convulsions if her fever increased in the afternoon, advised the use of a warm bath and the application of sinapisms to the extremities and spine if any symptoms of convulsions came on. At four o'clock, P. M., was called in haste to see her again, the messenger saying "she was threatened with fits." On arriving found her in following condition: Fever high, skin hot and dry, pulse full and quick, face very much flushed and head hot, with frequent starting and involuntary twitching of muscles. I asked her father if he had used the warm bath and mustard as I had advised in the morning? He said "no." I asked him why he had not—he replied thus: "Well, Doctor, I have seen those remedies used a great many times in fits, and have never seen them do any good yet." The answer struck me with a good deal of force and brought to mind a case I have seen about a fortnight before, very similar to the one then before me, in which the same remedies had been used with effects so evidently injurious that I had between the period of the two cases, more than once asked myself the question—is it correct or judicious practice to use such remedies, especially the sinapisms, in a disease where the nervous system is already irritated and excited to so dangerous a degree? But not having satisfied myself fully on the question, and they, together with cathartics and anti-spasmodics being the stereotyped remedies in such cases, I had advised their use in the morning in this case, for being young in the profession, I did not feel at liberty to depart from the rules laid down by those older than myself. Feeling satisfied I had to treat a case full of danger, and that the remedies in use is such cases were often valueless, if not *positively injurious*, I called up as quickly as possible my whole store of knowledge of the Materia Medica, to see if I could find a remedy suited to the case. I wanted a *nervous sedative free from narcotic effects*—where could I find it, or was there a medicine in the whole catalogue possessing such an effect.

A year or two before this several writers had advocated, the theory that quinine in *large* doses was a sedative. Would it produce the effect I desire in such a case as this, or would it be safe to give it with a *high fever* and a, *hot dry skin?* These were questions I could not answer, for I had been taught to give it *only* when the patient was *clear of fever* and in *small doses*, and had acted accordingly. I determined, however, to try it, and at once gave 4 *grs.* mixed with the same quantity of blue mass, (the oil given in the morning not having acted,) taking *good care* not to let her father know what I was giving, for fear he would objeet to her taking quinine with so high a fever. In taking it, she lost about one-fourth the dose. Scarified her gums again and had cloths wrung out of cold water, kept constantly to the head. The interest with which I watched its effects may be easily imagined, never before having given it in fever, or in such a dose to one so young. At 6 o'clock, about one hour and a half after the first dose my little patient was sleeping, with very little twitching or starting and the surface of the body becoming moist. The effect appearing to be salutary, I determined to repeat the dose, giving as before, 4 grs. aa quinine and blue mass. At 7 o'clock she was sleeping *soundly* and *quietly*, the surface bathed in a *profuse perspiration*—the heat of head very much diminished and the pulse shower and softer. I therefore concluded to wait an hour before giving another dose. Before the hour expired; she cried for something to eat. At 8, gave 2 grs. quinine and directed an enema, which acted well. At 9, scarified gums again and left for home, leaving three powders of quinine, 2 grs. aa, to be given at 10 P. M., and 2 and 6 A. M. the following—she was still perspiring, her fever much lessened and entirely clear of spasmodic symptoms.

9th. 8 A. M., my patient sitting on the floor at play, entirely clear of fever, having slept well through the night. Fearing a return of fever in the afternoon. divided 10 grs. quinine in three powders and directed them to be given at 9, 12 and 3 o'clock, and to be informed if she had any return of fever.

10th. Mr. K. came in and reported Julia better, having had no fever the day before. Still fearing a return of fever, divided 8 grs. quinine in three powders and directed them given as on the day before, after which I heard nothing further from the case.

Case 2. Dentition with convulsions. Was requested on the morning of Aug. 15th, 1847, by Mr. S. to prescribe for his son John, aged 13 months, who, he said had fever. At 1, o'clock, P. M., received a message from him requesting me to visit his son as quickly as possible as he had convulsions, I reached his house at 2 P. M., (it being six miles in the country,) and found the child with high fever, skin hot and dry, face flushed, head hot and pulse quick and full, he was sleeping, but frequently distured from any slight noise. Learned he had had two convulsions, each lasting from 10 to 20 minutes—bowels open from the medicine prescribed in the forenoon. On attempting to examine his gums, he was taken with a convulsion, which lasted about 15 minutes. As soon as it subsided, finding his gums swollen, I scarified them and gave 6 grs. sulphate quinine, and repeated the dose in one hour. One hour after the dose, he was *sleeping quietly and perspiring freely*. Directed 3 grs. more to be given at 6 o'clock, and if there should be any starting, or other symptoms of convulsions, to give three grains at 10 P. M.

16th. Mr. S. came in and reported his child as having had a slight convulsion a few minutes before 6 on the previous afternoon. As soon as it subsided, gave the quinine, and at 10, the last dose. Said he had slept well during the night and was then clear of fever and very lively. Prescribed more quinine to be taken during the day, but have forgotten the quantity. After which I heard nothing further from the case.

Case 3. Convulsions. Aug. 21st, 1857, 2 o'clock, P. M., was called to see Anna, a negress of light complexion, aged 12 months, the property of Dr. B., and found her in a convulsion, with bloody froth issuing from the mouth, which caused her mother to think she was choked. Her owners being from home, a lady living on an adjoing lot, had been sent for, and had put her in a warm bath before my arrival. The spasmodic action being so great as to prevent deglutition. I kept her in the bath and poured cold water on the head and down the spine. In a short time the convulsion subsided sufficiently to allow her to swallow—I gave her six grains quinine. The effort to swallow appeared to increase the convulsion and it continued unabated for at least half an hour, at the expiration of which time seeing no evidence of its passing off, and fearing she would die in it, I had 12 grs. quinine given by enema, which was retained. For at least 20 minutes the convulsion continued without any abatement. As soon as she could swallow, I gave her 6 grs. quinine by the mouth. During this time the warm bath to the lower extremities and cold water to the head and spine had been perseverin!y used. In one hour after taking the last dose, she was sleeping quietly and perspiring freely. Her bowels being open, gums not swollen, nor having eaten anything indigestable, I was at a loss to account for the cause of the attack—her mother, a very intelligent mulatto, said she was well and at play, when seized with a convulsion. The spasmodic action was so great I could not ascertain the condition of her circulation and being in the bath could not judge of the condition of her skin—her head felt very warm.

5, P. M. Sleeping, perspiring freely and clear of spasmodic symptoms.

6, P. M. Still asleep, but has been awake once—skin moist and free from all symptoms of nervous excitement.

22d. 8 o'clock, A. M. Found my patient in the yard at play with her nurse, clear of fever and learned she had slept quietly through the night. Case dismissed.

Case 4. Intermittent fever with Convulsions. September 6th, 1858, was called at 5 P. M. to visit Rose, aged 5 years, the property of Mr. W. Found her on my arrival in a convulsion, and learned she had had one about an hour before—her skin was hot and dry, tongue foul, pulse full and quick, but unable to count it, bowels constipated. About two hours before, her fever then not being very high, 6 grs. quinine had been given her. A warm bath being at hand, I had her placed in it, and kept there until the violence of the convulsion subsided, when I gave her 6 grs. quinine, in half an hour after which, she was sleeping and perspiring freely. Directed 3 grs. aa quinine and calomel, to be given at 6 o'clock and repeated at 9—a dose Ol Ricini and Ol Terebin. at 6 the next morning and to begin at 10 and give 4 grs. quinine every hour until 12 grs. should be given.

7th. 4 P. M. Found Rose clear of fever, had no return of convulsions during the night—the calomel and oil had acted well. Case dismissed.

The following case was first seen by my co-partner Dr. R. L Gamble, from whom I have obtained the following particulars:

Case 5. Intermittent fever with convulsion. October 2d, 1858, was called at 5, P. M., to visit Elick, aged one year and nine months, the property of Capt. P. Found him with high fever and learned he had been attacked two days before with chill—had had one on the morning of that day, followed by fever, which had increased during the afternoon. Prescribed and left. In two hours after, was called in haste, the messenger saying "Elick had a fit." On reaching the house, found the convulsion had passed off and Elick in the following condition: Pulse 150, strong and full—skin hot and dry, dozing, but frequently starting—gave 6 grs. quinine. In one hour after the second dose, pulse 110, skin cool and moist and sleeping more soundly. Directed 6 grs. quinine to be given in two doses through the night.

3d. 9, A. M. Visited Elick myself, wishing to note the effects of so large a quantity of quinine. Found his pulse 100 and skin soft and cool. He was evidently very fully quininized as could be seen from the bewildered expression of his countenance, though he answered questions correctly and did not appear very deaf—he had slept quietly through the night—bowels freely open. Desiring to keep up the quinine influence, gave him 3 grs., and directed 3 grs. to be given at 12 M., and if after that hour any spasmodic symptoms should be seen, to give him a dose of 6 grs., and inform me forthwith, but heard nothing from him until the morning of the 4th, when Dr. Gamble visited him and found him clear of fever—had had no symptoms of convulsions. Prescribed 3 grs. quinine at 10, and 3 grs. at 12 M. Case dismissed.

*Remarks.*—The above is more meagre in the number of cases reported than I would have liked, but not having kept a "case book," I have only been able to recall the circumstances connected with the first three cases treated with quinine, and the two occuring in September and October of this year. Since the occurrence of the first case, I have not in a single instance used mustard, being convinced it only increases, instead of diminishing nervous and vascular excitement—very seldom have I used the warm bath, quinine being the remedy upon which I have relied almost entirely, and an experience of twelve years, enables me to say of its use:

1st. That it is entirely safe. I doubt not that some will think the treatment detailed in the foregoing cases not only rash but hazardous, but I have yet to see the first injurious effect resulting from it. I am not ignorant of the fact that death has resulted from an over dose of quinine—the same is true of opium, and yet who would hesitate to administer in certain conditions of the system demanding its use, a quantity of the latter, which would probably prove fatal in health, for it is in disease, not in health, that we are to judge of the effects and doses of medicine. The same rule applies to quinine, that does to opium, and it is only in that condition of the nervous system which we see in convulsions, that I would think of giving it in such doses to a child, in any other condition I should fear death would be the result. Its general effects are such as here described, first a quiet sleep, quickly followed by sweating, more or less profuse, from which the child awakes free from spasmodic symptoms, and either clear of fever, or with the fever very much lessened, if as usual in most cases, it had fever before the convulsion. And this leads me to speak briefly of the remedy as a sedative Is quinine a sedative? Like all other questions, this is one to which there are two sides. Prof. Wood, U. S. Dispensatory, says: "From its occasional effect in diminishing the frequency of the pulse and the general strength, it has been supposed to be essentially sedative in large doses. Such an opinion, unless well founded, might lead to hazardous practice. The probability is that the apparently sedative effect upon the circulation arises from an overwhelming stimulant influence upon the cerebral centers, whereby the system is deprived of the support of these centers, and the heart's action is depressed with other organic functions. Similar effects may be obtained from excessive doses of most of the cerebral stimulants. * * * * In the present state of our knowledge. therefore, it is safest to consider sulphate of quinine as a direct and powerful stimulant to the brain." On the other hand, Prof. Dunglison, "New Remedies," says: "This decisive sedative action has been observed by the author over and over again, when the sulphate of quinine has been given in free doses," and cites the names of near a dozen writers who express the same opinion. Pereira, Mat. Med. and Therap. expresses no opinion on the subject, merely observing: "In some instances it has appeared to act as a stimulant, in others as a sedative." My experience leads me to look upon it as being a very decided sedative, and this effect, I think, is directed more to the nervous than the vascular system, for as an arterial sedative, it will not compare with Veratrum Viride, nor have I ever seen the same tranquilizing effects following the use of veratrum that I have almost invariably observed from the use of quinine. A part of this tranquilizing effect may be due to a narcotic property, for in large doses, it appears to be somewhat narcotic, though very slightly so, for I have never observed anything approaching a deep narcotism from its use, even in larger quantities than was given in either of the foregoing cases.

2d. It is effectual. Before adopting the quinine treatment, it had been my misfortune to lose cases of convulsions, and it was "with fear and trembling" that I visited them—now I feel as much confidence in relieving, and as little dread in treating them, as I do a case of fever, and can say with candor that I have lost but one case during the last twelve years, nor do I now remember but three cases in which there was a recurrence of the convulsion after the system became fully impressed with the remedy. A very large proportion of the cases we are called to in this section of the State occur in connection with malarial fevers—apart from its sedative effect, what remedy so potent for the cure of these fevers, as quinine, and by treating the convulsions with it, we are apt to find in checking the one, we have at the same time cured the other. The amount of febrile excitement in a case, is a question of no moment, so far as the use of quinine is concerned, provided convulsions or even symptoms of them be present. I never wait for the fever to cool, but proceed at once to administer it, *and the higher the fever, the larger the dose I give.* The condition of the nervous system present in convulsions, appears to render the stomach more tolerant to its use than it is, in most diseases, for I have seldom been annoyed by having it rejected, as is freqnently the case, in giving it to children. If the bowels are not open, I give it in combination with some cathartic, aud hasten the action of the latter, when desirable, with enemas. In cases produced by dentition, errors in diet, intestinal irritation, &c., measures should be taken to remove the exciting cause, if possible; after that is done, the nervous system is generally left in a highly excited and irritable state, and the little patient is apt to have one or more convulsions before they entirely cease—in that condition of the system, quinine allays the excitement and prevents a recurrence of the convulsion with more certainty than any remedy with which I am acquainted.—*Savannah Jour. of Med.*

